# The proteomic content of *Varroa destructor* gut varies according to the developmental stage of its host

**DOI:** 10.1371/journal.ppat.1012802

**Published:** 2024-12-30

**Authors:** Vincent Piou, Karim Arafah, Michel Bocquet, Philippe Bulet, Angélique Vétillard

**Affiliations:** 1 Centre de Recherche sur la Biodiversité et l’Environnement (CRBE), UMR5174, CNRS-Université de Toulouse III-IRD, Université Paul Sabatier, Toulouse, France; 2 Plateforme BioPark d’Archamps, Archparc, Archamps, France; 3 Apimedia, Pringy, Annecy, France; 4 CR Université Grenoble Alpes, Institute for Advanced Biosciences, Inserm U1209, CNRS UMR 5309, Grenoble, France; 5 Conservatoire National des Arts et Métiers (CNAM Paris), Unité Métabiot, Ploufragan, France; University of Kwazulu-Natal, SOUTH AFRICA

## Abstract

The nutritional physiology of parasites is often overlooked although it is at the basis of host-parasite interactions. In the case of *Varroa destructor*, one of the major pests of the Western honey bee *Apis mellifera*, the nature of molecules and tissues ingested by the parasite is still not completely understood. Here, the *V*. *destructor* feeding biology was explored through artificial feeding, dissection of the mite’s gut and proteomic analyses. More specifically, the proteome of guts extracted from starved mites and honey bee-fed mites was compared to highlight both the parasite proteins likely involved in food processing and the honey bee proteins actually ingested by the mite. We could identify 25 *V*. *destructor* candidate proteins likely involved in the parasite digestion. As the host developmental stages infested by the mite are diverse, we also focused on the identity and on the origin of honey bee proteins ingested by the mite when it feeds on larvae, pupae or adults. We highlighted profiles of consumed honey bee proteins and their variations throughout the *V*. *destructor* life cycle. These variations matched the ones observed in the honey bee hemolymph, showing that this tissue is an important part of the mite’s diet. Based on the variations of abundance of the most consumed honey bee proteins and on their functions, the potential implication of these key candidate nutrients in *V*. *destructor* reproduction is also discussed.

## Author summary

*Varroa destructor* is one of the major parasitic pests in modern beekeeping worldwide. Since it shifted host from Eastern to Western honey bees, it was shown to weaken colonies by feeding on both immature and adult stages while transmitting several deadly viruses in the process. Nutrition, an overlooked aspect of parasite biology, is thus a key to comprehend the *V*. *destructor* life cycle and its impacts on its honey bee host. We explored the feeding physiology of this ectoparasite by analysing the protein content of its isolated gut to compare it with the protein composition of the honey bee tissues ingested. We highlighted several mite proteins probably involved in digestion and many honey bee derived proteins acquired during the feeding. Honey bee hemolymph is an important part of the mite’s diet although the diet could vary throughout the cycle, especially when mites feed on adult bees. The abundance of the most regularly ingested honey bee proteins such as Vitellogenin or Hexamerin varies throughout the bee development and could directly impact the parasite physiology. The analysis and identification of key proteins required for the mite’s survival and reproduction will pave the way for the development of more specific control strategies.

## Introduction

The study of parasite interactions with their animal host often focuses on the impact of host nutrition on parasites’ survival or behaviour [1]. The nutrition of the parasite, on the other hand, tends to be overlooked or limited to the damage it causes to the host. As in any other species, the diet and feeding biology of a parasite is crucial to obtain the necessary nutrients for reproduction and survival [2–4]. Identifying the host tissues consumed and the molecules ingested when the parasite feeds is thus a first step towards a better understanding of its physiological needs and ecology [5]. This is especially important when the host species has an essential environmental or agroecological role.

With hundreds of crop plants and agricultural products depending on honey bee pollination, the Western honey bee *Apis mellifera* is a perfect example of such essential insect species [6,7]. As other eusocial insects, honey bees suffer from many parasites and pathogens that benefit from their social organisation to spread within colonies [8]. In the past few decades, *Varroa destructor* became the major ectoparasite of Western honey bees [9,10]. Since this native pest of the Eastern honey bee (*A*. *cerana*) shifted host, it has caused damage to *A*. *mellifera* colonies in the Northern hemisphere [11]. The impact of the parasite is both direct through the parasitization of honey bees, and indirect through the transmission of deadly viruses [12–14]. *Varroa destructor* females infest and feed on adult bees during the dispersal phase and on juvenile stages, namely larvae and pupae, during the reproductive phase. Regardless of the host developmental stage being parasitized, it was thought that the mite fed solely on honey bee hemolymph until a study showed that the adult fat body was also part of the parasite diet during the dispersal phase [15]. More recently, the work of Han and colleagues focused on the honey bee hemolymph proteins found inside the whole body of *V*. *destructor*. The diet of the mite would in fact change from fat body to hemolymph between the dispersal and the reproductive phase [16], as suggested by the survival of mites fed only on honey bee larva hemolymph [17]. The change in diet composition throughout the mite life cycle is of prime interest as it could in fact play a critical role in the mite’s physiology. The protein intake from the diet is indeed considered essential in the reproduction of arthropods [18]. In the case of *V*. *destructor*, in addition to the classic protein intake, females seem able to directly use their undigested host proteins to improve the energetic stocks needed for the reproduction [19–21]. Several specific honey bee proteins obtained through feeding on its pupal or adult host are thus crucial in the parasite metabolism [19,21]. The exploration of the honey bee proteins ingested throughout the parasitic life cycle could shed light both on key nutrients required by this major ectoparasite, on their origin in the host body and on their function.

Mass spectrometry-based proteomics has emerged as a powerful tool to study the life cycles of parasites such as ticks [22], nematodes [23], or protozoa [24]. In the case of *V*. *destructor* nutrition, the use of proteomic analyses allowed Han and colleagues to compare the honey bee hemolymph proteins that are found in mites sampled during dispersal on adults and reproduction on pupae. They highlighted that there was a clear change of protein profiles between pupa- and adult-fed mites and attributed this change to the type of tissue consumed by the mite, namely hemolymph on honey bee pupae or fat body on adults. However, proteomic data coming from the honey bee fat body are still lacking and could not be included in this first analysis. Furthermore, honey bee larva-fed mites should also be considered as this host developmental stage is crucial for the mite’s reproduction activation [25]. Our aim was thus to gain a better understanding of the ectoparasite nutrition in terms of tissue consumed and diet requirements during its development. Contrary to previous studies, we focused on the proteome from mite’s isolated gut as it is the major organ involved in nutrition and digestion in the parasite. Furthermore, the inclusion of artificially starved mites was used as a baseline both for the presence of bee proteins and of *V*. *destructor* proteins inside mites’ guts. The presence of such control conditions is informative and many studies about parasite nutrition use starved or partially fed individuals as negative controls [26–30]. By comparing these baseline levels to proteomes from mites fed on honey bee larvae, pupae or adults, the identification of the most frequently ingested host proteins was possible as they appear in higher quantities compared to starved mites. Similarly, putative digestive *V*. *destructor* proteins are mobilized as soon as the parasite ingests honey bee tissues and are expected to be up-regulated in honey bee-fed mites compared to starved mites. Through artificial feeding and off-gel bottom up proteomics, we thus zoomed in on the proteomic profiles from the guts of mites fed on *A*. *mellifera* specimens at different developmental stages (larvae, pupae, and adults). The proteomic data obtained from *V*. *destructor* guts were further compared to results of proteomic analyses directly ran on both honey bee tissues putatively consumed (i.e. hemolymph and fat body). The honey bee proteins found inside *V*. *destructor* guts are indeed expected to be related to the protein profile from the consumed honey bee tissues. This could further shed lights on the origin of the proteins actually ingested by the mite for each of the three developmental stages selected and on the nutritional variations throughout the parasite life cycle. The nutrients available are indeed expected to vary between the honey bee developmental stages, which could in the end impact the physiology of the mite during its crucial reproductive phase.

## Results

### Identification of *V*. *destructor* proteins putatively involved in the mites food processing

**Fig 1:**
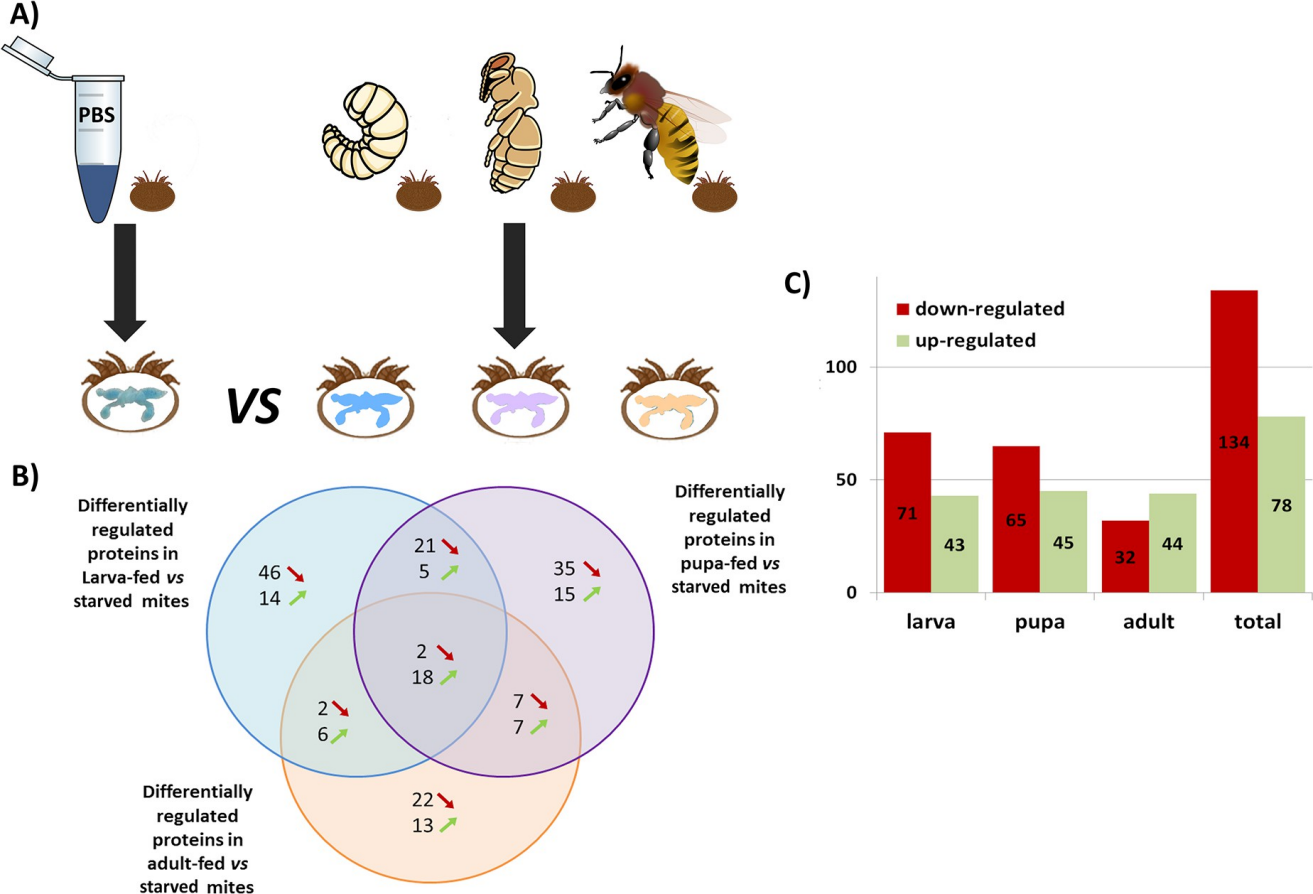
Exploration of the *Varroa destructor* gut proteomic profiles between fed and starved mites. (A) Experimental groups compared in the gut proteomic analysis of Acari proteins. Mites naturally fed on larvae, pupae or adults for 24h were compared to control starved mites that only had ingested PBS. PBS had to be added in the starved condition as *V*. *destructor* dies from desiccation under 24h if deprived of a water source [44], (B) Venn diagram showing the common number of differentially regulated Acari proteins between larva-fed and starved mites (in blue), between pupa-fed and starved mites (in violet) and between adult-fed and starved mites (in orange). The green and red arrows represent up- and down-regulation, respectively. (C) Amounts of up- and down-regulated proteins in larvae, pupae, adults or in total. For each group, analyses were run on three replicates of 4 mites’ gut pooled together (N = 3 pools of four mite’s gut per condition). Larva (https://doi.org/10.7875/togopic.2022.304) and pupa pictures (https://doi.org/10.7875/togopic.2022.305) were retrieved from the DataBase Center for Life Science and were conceived by Haru Sakai and Hiromasa Ono.

A total of 1,847 Acari proteins could be identified in the extracted guts of adult females *V*. *destructor* in our study (S1 Fig). The measurement of the protein abundances and subsequent calculated ratios across feeding conditions were performed using the label-free quantitative (LFQ) mass spectrometry (MS proteomics strategy). Based on these ratios, we highlighted specific Acari proteins differentially regulated (i.e. superior to 2 or inferior to 0.5) when mites were starved compared to mites that fed on their honey bee larval, pupal or adult hosts (Fig 1). In our experimental conditions, a tendency towards down-regulation was detected as approximately 63.2% (134/212) of the Acari proteins detected were down-regulated in honey bee fed mites when compared to starved mites, while 78 (36.8%) proteins were up-regulated (Fig 1). This tendency changed when only considering Acari proteins significantly different in all three categories (larva, pupa or adult fed mites) compared to starved mites. Indeed, 18 Acari proteins were up-regulated in all three categories and only two were always down-regulated (Fig 1 and S1 Table). In addition, an Endochitinase-like protein (XP_022673141.1) not listed in the 18 proteins from Fig 1 was up-regulated in all categories but the larva-fed *vs* starved factor was slightly inferior to 2 (S1 Table). Four proteins were also differentially regulated in all three categories but varied in different directions (the Ankyrin repeat domain containing protein 13 A0A132A0G5, the Chorion peroxidase T1KYK0, the Aldo_ket_red domain-containing protein T1JXC1 and the uncharacterized protein XP_022662294.1 in the S1 Table).

In this reduced pool of mostly up-regulated Acari proteins, the most represented functions were transport or secretion (7/25) with proteins like Vacuolar protein sorting-associated protein 28 homolog, solute carrier family 15 member 1-like protein, Rab GDP dissociation inhibitor, signal recognition particle subunit SRP68-like, FGGY carbohydrate kinase domain-containing protein-like isoform X1, Vesicle-fusing ATPase or Inositol-3-Phosphate synthase [29,31–37]. Immunity or stress response (5/25) were also well represented with Thioredoxin-dependent peroxide reductase-like protein, Solute carrier family 15 member 1-like protein, protein LSM14 homolog A-like, Natterin-4-like and Chorion peroxidase [31,32,38–43] (S1 Table). Furthermore, more than half of the proteins identified (16/25 or 64%) have already been detected in previous studies focusing on the gut or on the feeding status of other ticks, mites or hematophagous insects (S1 Table).

The proteomic exploration of the mite gut content also gave us access to many host-derived proteins ingested by the mite.

### Focus on the honey bee proteins ingested by the mite

#### Starved mites as a baseline to identify ingested host proteins

After 24h, the starved mites survival reached 77.6% [CI95: 65.8–86.9]. This percentage dropped to 32.8% [CI95: 21.8–45.4] after 48 hours, preventing a longer stay on this artificial medium (S2 Fig). Regarding proteomic analyses, a total of 560 *Apis spp*. proteins putatively coming from the ingested host tissues could be identified inside the extracted guts of *V*. *destructor* females (S1 Fig). Among them, 106 (18.9%) could still be found in the starved parasite gut after 24h without any contact with the host (S1 Data). However, the number of different *Apis* spp. proteins characterized in the mite’s gut was lower in starved mites when compared to larva-, pupa- or adult fed-mites. The abundance of proteins showed the same trend when compared to the starved control mites and a clear tendency towards over-representation of *Apis* spp. proteins was observed in honey bee fed groups. More precisely, 90.4%, 86.1% and 92.3% of the differentially expressed honey bee proteins were more abundant in larva-, pupa- and adult fed mites, respectively, than in starved mites (S2 Table).

#### Richness of honey bee proteins in mites guts and correspondence with the proteomic profile of honey bee hemolymph

The profiles of *Apis* spp. proteins found inside *V*. *destructor* guts were different when the mite had fed on juvenile stages (spinning larvae or pink-eyed pupae) or on adults (S3 Fig). On the contrary, pupa- and larva-fed mites shared closer *Apis* spp. protein profiles.

**Fig 2:**
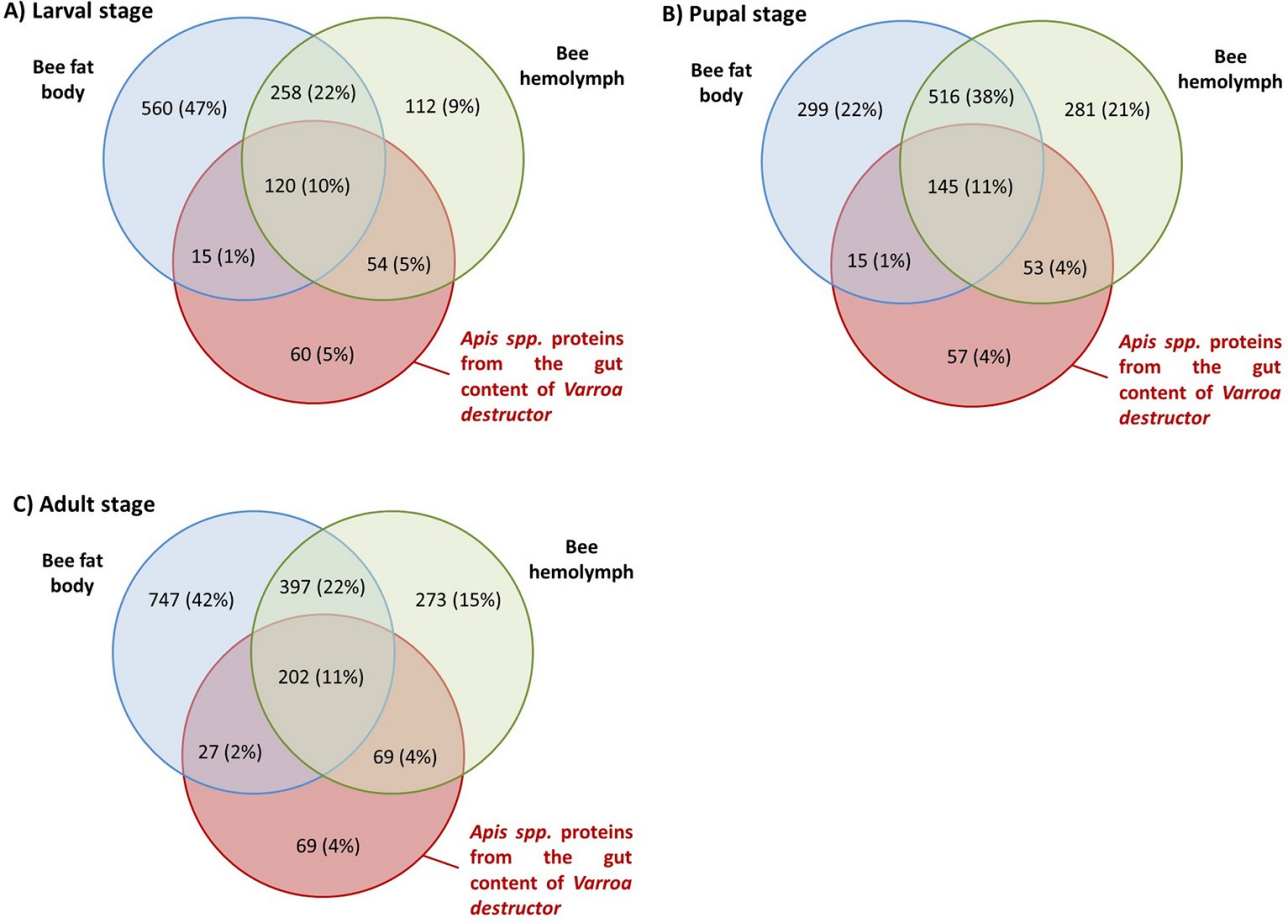
Venn diagrams of *Apis* spp. proteins found in both *V*. *destructor* guts and honey bee hemolymph or fat body. (A) Venn diagram of *Apis* spp. proteins shared between mites that fed on spinning larvae for 24h and spinning larvae fat body or hemolymph; (B) Venn diagram of *Apis* spp. proteins shared between mites that fed on pink eyed pupae for 24h and pink eyed pupae fat body or hemolymph.; (C) Venn diagram of *Apis* spp. proteins shared between mites fed on one day old adult honey bees for 24 hours and one day old adult fat body or hemolymph. (N = 3 pools of four mite’s gut per condition and three pools of three honey bee hemolymph or fat body per condition).

We further compared the *Apis* spp. proteins found in the *V*. *destructor* gut to the proteins found in honey bee hemolymph and fat body. These comparisons were performed for each of the three developmental stages on which the parasite had fed (spinning larvae, light pink-eyed pupae and emerging adults). Naturally, a high number of *Apis* spp. proteins was found in honey bee tissues, especially in the fat body. Depending on the honey bee developmental stage, 189 (larval stage), 213 (pupal stage) and 298 (adults) of these *Apis* spp. proteins from honey bee tissues were also found in the mite’s gut (Fig 2).

Even though a high proportion of the common proteins were shared between hemolymph and fat body, some were specific to one honey bee tissue. Regardless of the honey bee stage, the number of proteins in common between *V*. *destructor* gut and the honey bee hemolymph is higher than the number shared between the mite gut and the honey bee fat body (Fig 2). More precisely, a total of 54, 53 and 69 proteins from the parasite’s gut are specific to the honey bee larval, pupal or adult hemolymph against 15, 15 and 27 proteins for the honey bee fat body, respectively.

To further analyse the origin of these *Apis* spp. proteins found inside the mite’s gut, we ran generalised linear models (GLMs) to relate the protein counts in honey bee tissues and in mites’ gut. The counts correspond to the frequency of detection of each protein (classified by its accession number) in three biological replicates. Firstly, analyses of correlations show a general significant correlation between the *Varroa* gut content and the honey bee tissues (GLM Poisson: Hemolymph, Chisq = 1115.98; p<2.2e-16 ***/Fat_body, Chisq = 17.41; p = 3.01e-05 ***). However, regardless of the developmental stage of the honey bee on which mites had fed, the data from *V*. *destructor* gut was always more strongly correlated to the honey bee hemolymph than to the honey bee fat body (Tables 1 and 2 and S4 Fig). The relation between the protein content of the mite guts and of the honey bee fat body is even not significant in all cases but one. This observation was valid both when the entire list of *Apis* spp. proteins was considered to take the absence of proteins into account (Table 1) and when we focused only on the 189, 213 and 298 *Apis* spp. proteins shared between the mite and the honey bee tissues (Table 2). Multicollinearity risks were checked through the measurement of the variation inflation factor (VIF). Values were always inferior to 1.2 (far below the threshold of 5).

**Table 1:** Statistical analysis of the relation between all *Apis* spp. protein content from *Varroa* gut and all *Apis* spp. protein content from honey bee tissues. In this first analysis, the entire set of *Apis* spp. proteins was considered.

		Host proteins in guts of mites fed on different honey bee stages
Honey bee larva	Fat body proteome	Chisq = 8.80; p = 3.02e-03
	Hemolymph proteome	Chisq = 165.68; p<2,2e-16
Honey bee pupa	Fat body proteome	Chisq = 1.77; p = 0.18
	Hemolymph proteome	Chisq = 97.33; p<2,2e-16
Honey bee adult	Fat body proteome	Chisq = 1.86; p = 0.17
	Hemolymph proteome	Chisq = 394.45; p<2,2e-16

**Table 2:** Statistical analysis of the relation between shared *Apis* spp. protein content from *Varroa* gut and shared *Apis* spp. protein content from honey bee tissues. In this second analysis, only proteins in common between *V*. *destructor* gut and honey bee tissues were taken into account.

		Gut contents of mites fed on different honey bee stages
Honey bee larva proteome	Fat body	Chisq = 0.0045; p = 0.95
	Hemolymph	Chisq = 12.15; p = 4.904e-04
Honey bee pupa proteome	Fat body	Chisq = 0.04; p = 0.83
	Hemolymph	Chisq = 17.70; p = 2.59e-05
Honey bee adult proteome	Fat body	Chisq = 0,39; p = 0.53
	Hemolymph	Chisq = 20.34; p = 6.49e-06

#### Identification and abundance of the most frequently ingested honey bee proteins

When focusing on the presence or absence of proteins, only 54 to 77 proteins are consistently found in the three biological replicates depending on the developmental stage considered (S2 Table). Several proteins related to energy storage or transport, such as Transferrin, Hexamerin, Vitellogenin, Apolipophorin and Larval-specific very high-density lipoprotein appeared in all our samples from larva, pupa and adult fed mites. In addition, besides enzymes and proteins from the extracellular matrix, Odorant binding proteins (OBP 13–15, 18) also appeared in all samples.

**Fig 3:**
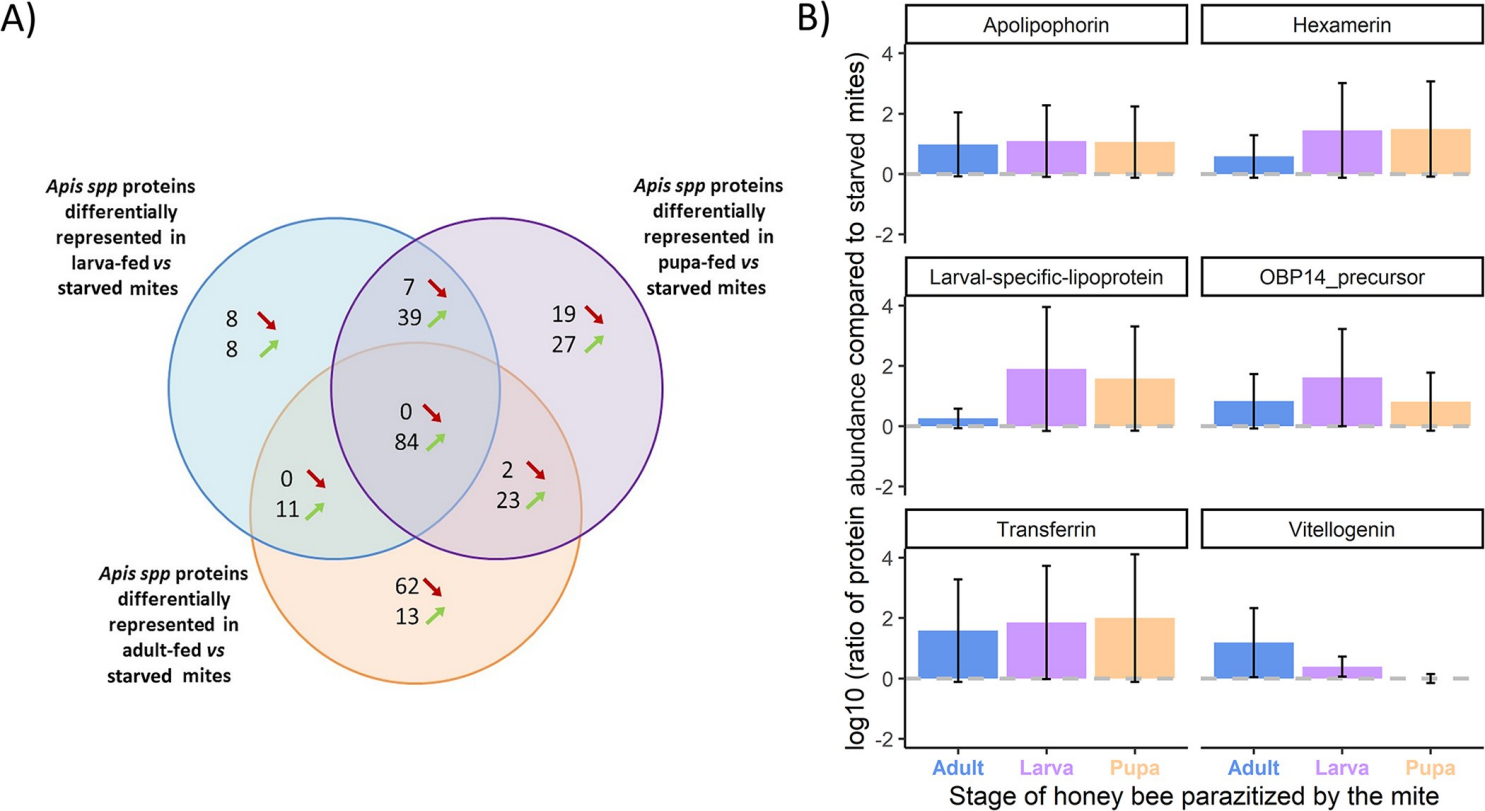
Comparison of protein abundances between starved controls and honey bee-fed mites. (A) Venn diagram of *Apis* spp. proteins differentially represented in the gut of honey bee-fed mites compared to starved controls that ingested PBS. (B) Barplots representing the log10 ratios of 6 of the most frequently over-represented proteins compared to the same proteins in the starved group of mites. Namely, the log10 ratios of Apolipophorin (XP_026298285.1), Hexamerin 70b precursor (NP_001011600.1), Transferrin (AAO39761.1), Vitellogenin precursor (NP_001011578.1), Larval-specific very high-density lipoprotein precursor (NP_001318046.1) and Odorant binding protein 14 precursor (NP_001035313.1) taken as an example for OBPs, are represented here. The grey dashed line represents the threshold between over- and under-representation (N = 3 pools of four mite’s gut per condition).

Many of these proteins present in all three biological replicates are also more abundant in bee-fed than in starved parasites, whether they infested larvae, pupae or adults (S2 Table). Transferrin, Vitellogenin, Hexamerin, Larval-specific very high-density lipoprotein, Apolipophorin, and several different OBPs (OBP3, 13–15, 17 and 18) belong to the list of 84 proteins over represented in all guts of mites fed on honey bees when compared to starved mite (Fig 3A and S2 Table). These proteins are thus always ingested in high quantities except for honey bee Vitellogenin in pupa-fed mites (Fig 3B and S2 Table). The peak of expression of Vitellogenin in fact seems to be at the adult stage, which leads to high concentration within the mite gut after feeding. As shown in Fig 3B, the over-represented *Apis* spp. proteins found inside the mite’s gut do not only vary in comparison to the starved group but also between mites fed on different honey bee stages. The variation of abundance in the mite guts could reflect the same fluctuation in the ingested honey bee tissues.

#### Matches between abundance variations of *Apis* spp. proteins in mite guts and in honey bee tissues

**Fig 4:**
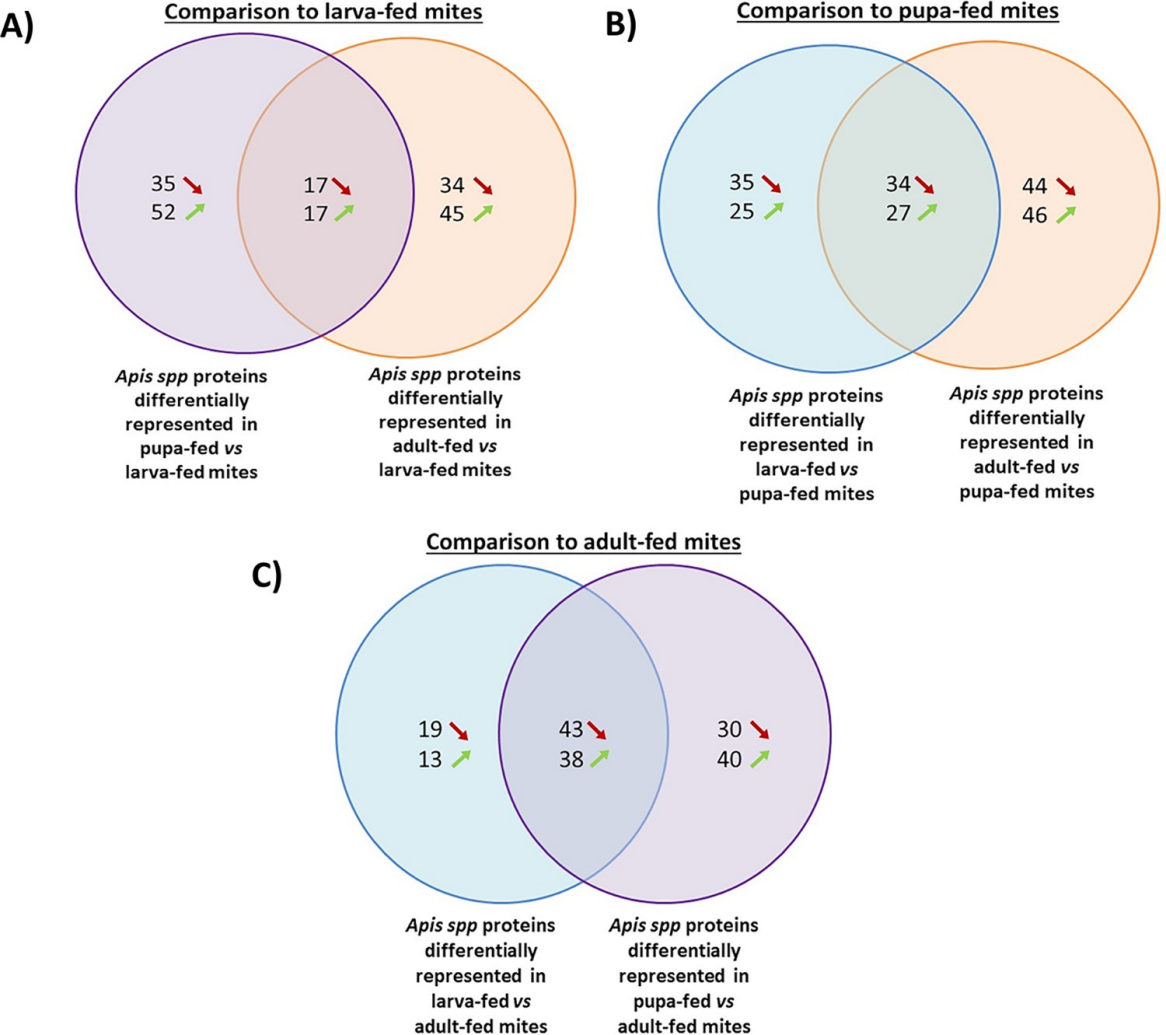
Venn diagrams showing the numbers of differentially represented *Apis* spp. proteins in guts of mites from the three honey bee-fed groups. Abundance ratios were compared to mites that fed on (A) honey bee larvae, (B) honey bee pupa or (C) one day old adult workers (N = 3 pools of four mite’s gut per condition).

The protein abundance ratios were calculated between the three following groups: (1) larva- and adult-fed, (2) adult- and pupa-fed, and (3) larva- and pupa-fed. We focused on significant ratios, i.e. on *Apis* spp. proteins that vary significantly according to the honey bee stages the mite fed on. On a total of 321, 326 and 306 proteins compared between two groups of mites fed on different host stages, around 40% varied significantly (Fig 4 and S2 Data). More precisely, 113 (Fig 4A orange circle) varied significantly between larva- and adult-fed mites; 151 (Fig 4B orange circle) varied significantly between adult- and pupa-fed mites, and 121 (Fig 4B blue circle) varied significantly between larva- and pupa-fed mites.

**Fig 5:**
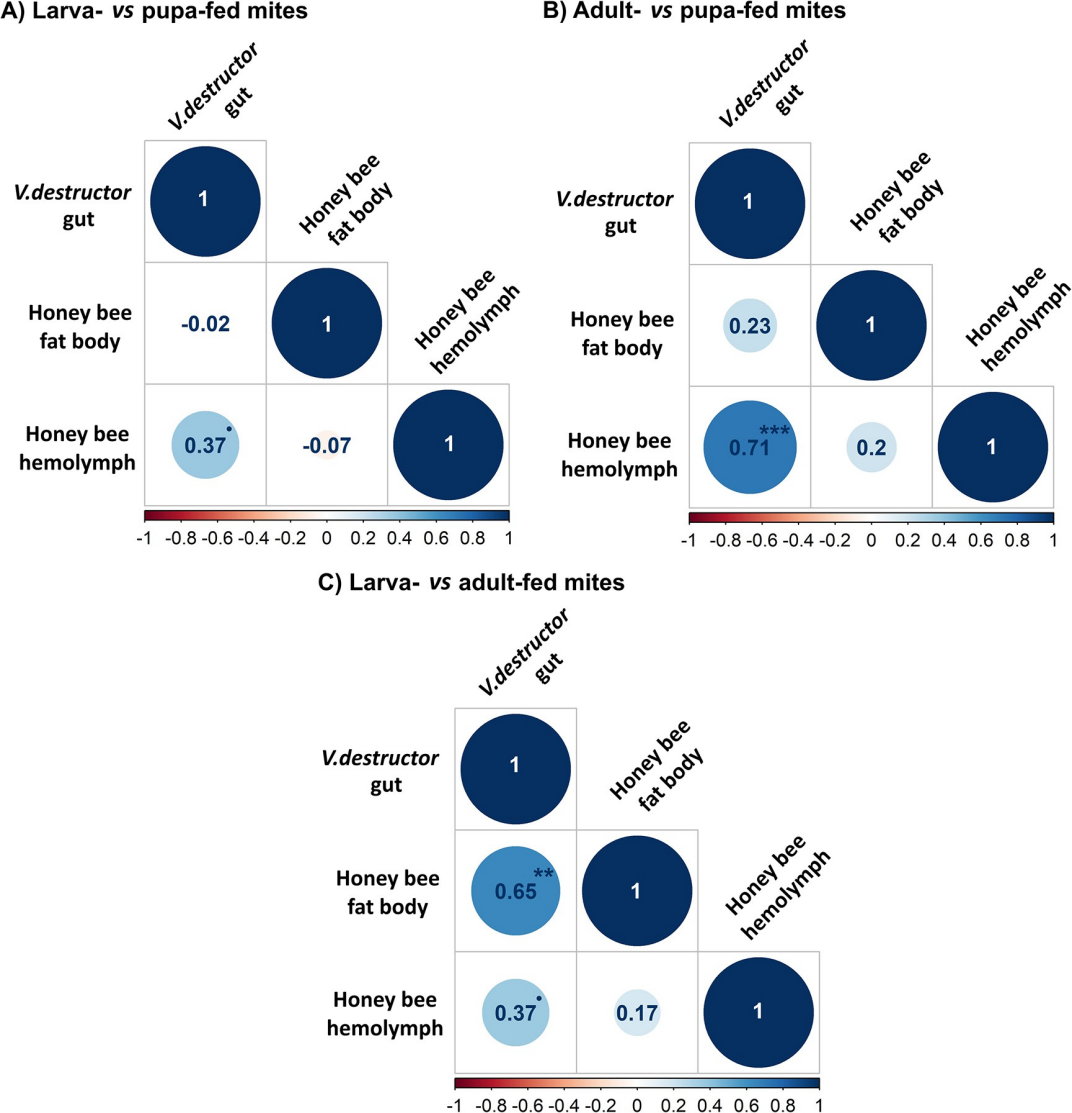
Correlations between the *Apis* spp. protein ratios of abundance in honey bee tissues and in *V*. *destructor* guts. (A) Abundance ratios of larva- *vs* pupa-fed mites; (B) Abundance ratios of adult- *vs* pupa-fed mites; (C) Abundance ratios of larva- *vs* adult-fed mites. Ratios between honey bee stages were calculated for honey bee hemolymph, honey bee fat body and *V*. *destructor* guts. The colour scale and the numbers inside the circles indicate Kendall τ and the asterisks show the level of significance from a general Kendall correlation after Benjamini-Hochberg correction (*** = <0.001;** = <0.01; * = <0.05; 0.05<•<0.10; N = 3 pools of four mite’s gut per condition and three pools of three honey bee hemolymph or fat body per condition).

The same ratio calculation and protein selection was applied to the honey bee tissues to be able to correlate the changes in honey bees and in the mite guts. In either hemolymph or fat body, only *Apis* spp. proteins that significantly vary in quantity throughout the honey bee cycle were retrieved to limit potential artefact when analysing covariations. Among these proteins, between 22 and 25 also vary significantly in the mite gut content. The covariations of these significantly fluctuating proteins were analysed and showed significant correlations between the honey bee tissues and the parasite gut content (Fig 5). As in the case of protein presence/absence analyses, the overall correlation was stronger between the honey bee hemolymph and the mite gut content (Kendall τ = 0.52, z = 5.46, adjusted-p<0.001) when compared to the honey bee fat body (Kendall τ = 0.30, z = 3.19, adjusted-p<0.01; S5 Fig).

This observation is also valid for the larva-pupa ratios (hemolymph: Kendall τ = 0.37, z = 2.33, adjusted-p = 0.06; fat body Kendall τ = -0.02, z = -0.11, p = 0.91) and for the adult-pupa comparison (hemolymph: Kendall τ = 0.71, z = 4.08, adjusted-p<0.001; fat body: Kendall τ = 0.23, z = 1.37, adjusted-p = 0.22) where the correlation between the parasite gut content and the honey bee fat body is low and not significant. On the contrary, the tendency reverses for the larva-adult ratios as the mite gut content becomes significantly correlated to the -honey bee fat body (hemolymph: Kendall τ = 0.37, z = 1.97,adjusted-p = 0.07; fat body: Kendall τ = 0.65, z = 3.46, adjusted-p<0.01).

The variable proteins highlighted through quantification are detailed in the S3 Table. Among the proteins already identified, Hexamerin expressed a peak at the larval stage then the level decreased during the pupal stage to reach its minimum at the adult stage (Fig 3B).

## Discussion

By comparing gut contents of mites that fed on honey bee larvae, pupae or adults to control starved mites, we were able to identify 25 Acari proteins mostly up-regulated as soon as *V*. *destructor* fed on its host. We could also highlight a series of honey bee derived proteins that are always ingested in high quantities when the mite parasitizes its host. These proteins found in *V*. *destructor* gut vary throughout the parasite life cycle and are correlated to the developmental variations in honey bee hemolymph.

### On the use of starved mite as a baseline to identify ingested host proteins

The presence of a negative control is always informative although in the case of nutritional studies, it can be a complex condition to set up. Many studies focusing on parasite nutrition use starved or partially fed individuals as negative controls [26–30]. However, the 24h survival of *V*. *destructor* totally deprived of food and water is often low [44] which is not suitable for a negative control. In our study, the mites were thus artificially maintained on a source of water to avoid desiccation, which allows 77.6% of mites to survive for 24h without access to any source of nutrients. After such period, even though honey bee proteins can still be found in the digestive tract of the mite, the richness of proteins found in control starved mites is reduced compared to the bee-fed groups, which seems consistent with a starvation period. Nevertheless, bee proteins were still detected in starved mites, which makes the quantitative comparison between bee-fed mites and starved controls relevant. This comparison was used to further identify proteins over-represented after feeding which means they were surely ingested during the previous 24h. This is why the second part of the study mainly focuses on over-represented *Apis* spp. proteins found inside the mite’s gut. Finding higher quantities of these proteins inside honey bee fed mites is consistent with the fact that mites did ingest honey bee tissues during the 24 hours spent on honey bee larvae, pupae and adults.

The reason why proteins are still found after a starvation period remains unknown. We suggest that since females *V*. *destructor* were initially sampled from naturally infested pigmented pupae, traces of honey bee proteins coming from previous meals remain even after 24h. A study of the kinetics of absorption/digestion of honey bee proteins in the gut of *V*. *destructor* after a meal could bring further conclusions. In any case, a longer amount of time would likely result in less or no honey bee protein remaining inside *V*. *destructor*‘s gut. The problem is that this timing cannot be extended without facing higher mortality of control mites. Simply adding sugar to the solution could have allowed to keep mites away from bees for longer period (S2 Fig) but the ingestion of carbohydrates is also a source of bias and would not have been compatible with the search of *V*. *destructor* proteins potentially involved in food processing.

### Identification of *V*. *destructor* proteins putatively involved in the mite’s food processing

First, *V*. *destructor* proteins up- and down-regulated after a meal were retrieved from the comparison of the gut proteomic profiles of naturally fed and control starved mites. Based on the tendency towards protein down-regulation detected in our study in honey bee fed mites (*i*.*e*. up-regulation in starved mites), no general reduction of basal metabolism and protein synthesis could be observed in starved mites. On the other hand, when all three honey bee fed groups of mites were considered, we found 25 gut proteins that were always differentially regulated when mites fed compared to control starved mites that had only ingested PBS. Most of these proteins were up-regulated in all three groups that fed on honey bees, regardless of the stage. Transport was the most represented function, followed by immunity and stress response. The immunity and stress response proteins were mostly up-regulated in honey bee fed mites, which does not fit with a stress response to starvation, although it cannot be entirely excluded. Remarkably, the majority of proteins identified here had actually been highlighted in previous transcriptomic or proteomic studies in the gut, the salivary glands or the synganglion of ticks, mites and hematophagous insects, especially after they had fed on their host (S1 Table). Proteins from *V*. *destructor* sialome were also detected in our gut samples [45]. This could indicate either an ingestion of salivary secretions during the mite feeding or a broad location of these digestive proteins in mites’ organs. Among the differentially regulated proteins, Peroxiredoxins such as the Thioredoxin-dependent peroxide reductase or Chorion peroxidase are known to play a crucial antioxidant role after blood feeding in ticks [38]. In our dataset, we also regularly detected Heat shock proteins (HSPs), a family of proteins expressed in many biological processes to permit protein folding and transport, and cellular protection including anticoagulant purpose among other functions [46,47]. Because these proteins were mostly up-regulated inside the gut of honey bee-fed mites regardless of the stage and since many proteins were already identified in studies about Acari nutrition, we suggest that they might be involved in food processing in *V*. *destructor*. However, proteins involved in mite digestion are not necessarily differentially regulated and other mechanisms could be involved, especially since *V*. *destructor* is able to assimilate undigested honey bee proteins. Protease inhibitors from the honey bee itself could indeed be used by the parasite to control protein degradation and use honey bee proteins for its own physiology, as suggested by Tewarson and Jany (1982) [48]. The exploration of the feeding physiology of *V*. *destructor* and other parasitic Acari species could shed light on the role of many of these proteins in the future. More specific gene expression or gene knockdown studies along with investigations of protein activity in starved and fed mites could confirm the digestive function of the up-regulated proteins highlighted here.

### Focus on the honey bee proteins ingested by the mite: do protein identity and abundance variations fit with their origin in honey bees?

By comparing control starved and honey bee-fed *V*. *destructor* females, we were able to identify proteins ingested from the parasitized honey bee stages. Analyses run on proteins that we could detect in the honey bee fat body and hemolymph showed that the richness of proteins was greater in the honey bee fat body than in honey bee hemolymph (Fig 2), at least in the adult and larval stage. A great variety of proteins detected in the honey bee fat body was absent or undetected in our experimental conditions inside the mite gut after feeding. Some of these proteins can still bring further information on the tissue ingested by the mite. This is for instance the case of the honey bee Perilipins-like proteins at the adult stage which were detected exclusively in the honey bee fat body but not in mite gut samples (S1 Data). Even when the focus is made only on proteins that are found in common between the parasite and its host, the total of proteins shared is higher between mite guts and honey bee hemolymph than fat body. The occurrence of the proteins across *V*. *destructor* gut samples is also more correlated to the honey bee hemolymph than to the fat body. This result does not rule out the consumption of fat body since some proteins consumed by the mite are only shared with this tissue. This could however mean that hemolymph is ingested in non-negligible proportions by the parasite at the larval, pupal and adult stage. Our quantitative analyses also support this hypothesis as correlations were stronger between the abundance ratios of honey bee hemolymph and mite guts. Altogether, our results indicate that the ratio of hemolymph to fat body ingested varies depending on the host developmental stage. Hemolymph could indeed be a primary consumed tissue in larvae and pupae, which would corroborate recent results showing i) that *V*. *destructor* is perfectly able to survive for long periods when fed hemolymph from larvae and pupae [17] and ii) that the proteome of mites seems to differ when fed on adults compared to mites fed on pupae [16]. The change of diet proposed by Han is further supported by the closer proteome profile between pupa and larva-fed mites than between adult-fed mites and the two other categories in terms of honey bee ingested proteins (S2 Fig). Besides, the adult *vs* larva comparison was the only case in which the protein ratios were more correlated between honey bee fat body and *V*. *destructor* gut. All other analyses showed a higher correlation with honey bee hemolymph in our study, which could further support a change in the diet when the parasite feeds on adult stages. However, it has to be noted that in our study, one day old adults rather than nurses had to be used to enable the survival of mites under our laboratory conditions. Fat body tissues are less developed in emerging honey bees compared to nurses [49], and variations in their proteomic profiles are probable (S4 Table). This could affect the quantity and quality of proteins ingested by the mite in our study compared to mites naturally infesting nurses. In natural conditions, *V*. *destructor* is indeed known to be attracted to nurse bees over younger bees or foragers, which would allow it to have access to a fully developed fat body [15,16,50]. To complete the analysis of adult honey bee tissues conducted in our study (S4 Table), a comparison between guts of mites fed on one day old adults and on nurse bees could further shed light on this hypothesis.

Among the proteins likely ingested from hemolymph, a restricted group of honey bee proteins were systematically detected in our samples (S2 Table). They were also detected at higher quantities after a meal on the host when compared to starved mites that only ingested PBS (S2 Table). Among these proteins, transport and storage functions are well represented. Apolipophorins, Lipoproteins, NPC cholesterol transporter and OBPs are all involved in lipid or hydrophobic molecules transport at different scales [51–59]. Transferrins are also responsible for the transport of iron inside the insect body and thus related to many iron-dependent functions, among which reproduction. Similarly, Hexamerins and Vitellogenin are considered as storage proteins and are especially important for insect reproduction and pupal development [60–64]. Other proteins always detected in our samples included Beta-ureidopropionase, Catalase, Enolase, Fibrillin, Laminin and Phenoloxidase. These proteins are ubiquitous and their roles vary from detoxification and involvement in extracellular matrix to cell growth or immunity [65–69].

These 12 proteins could be nutritionally relevant to the mite as they represent a reliable source of amino acid available throughout the mite’s life cycle. They were also recently described in hemolymph-derived artificial feeding media allowing the mites to survive over two to three weeks [17]. Furthermore, five of these proteins are well described in the literature about *V*. *destructor* and are known to be crucial for the mite’s reproduction. After the pioneer work of Tewarson et al. 1982 [21] on Vitellogenin, Ramsey et al. 2022 [19] identified nine honey bee proteins that can be found in *V*. *destructor* reproductive organs and eggs. Apolipophorin and Lipoproteins, Transferrin, Hexamerin and Vitellogenin which appear stored in *V*. *destructor* and its eggs [19,21] are also omnipresent in our mite gut samples. Our results show that these energetically valuable proteins involved in *V*. *destructor* reproduction are always ingested by the female mite throughout its cycle, regardless of the stage it feeds on. These proteins could thus represent important nutrients required by the mite to complete its cycle, as previously suggested in the study about survival of artificially fed mites [17]. Studies about the survival of mites artificially deprived from these proteins would confirm their importance in the parasite physiology.

In addition to being reliable nutrient sources throughout the parasite life cycle, the quantitative variations observed between the honey bee developmental stages deserve to be investigated. Ingesting different quantities of such proteins at precise moments of the cycle could trigger physiological processes such as reproduction [70]. The 5^th^ instar larval stage is for instance known to emit specific cues essential to the reproduction activation in *V*. *destructor* [25]. Some of the ingested proteins have the potential to be key physiological signals for the mite or at least to be nutritionally required for egg maturation once key signals are detected. Hexamerin concentrations seem to go through a peak at the larval stage. This peak of concentration is also observable inside the mite’s gut and would coincide with a crucial activation step during the reproductive phase for the parasite [9,10]. A similar observation can be done for the Vitellogenin at the adult stage. Adult honey bees could indeed be a primary source of Vitellogenin during the dispersal phase, which could trigger the oogenesis of female mites before they invade a larval cell [15]. In our samples, the peak of Vitellogenin is observed in adults and more specifically in older nurse-like honey bees (S4 Table). The dispersal phase on adults could thus represent an essential step of the mite’s reproduction, although experiments have led to divergent conclusions on its importance in the following parasite reproduction [50,71,72]. In addition to the omnipresent proteins highlighted in our study, some proteins shared between two stages only or always present at a specific stage but not detected at other times during the honey bee development are interesting. These proteins which are listed in S2 Table would worth further study as they could also be used as signals by the mite. In any case, the precise path and role of the host derived proteins for the parasite still have to be explored, for example through the monitoring of the kinetic of absorption after a meal or through the study about the effects of artificial depletion of candidate nutrients on the mite’s reproduction.

Altogether, by focusing on isolated *V*. *destructor* gut and comparing with proteomic data from honey bee fat body and hemolymph, our results bring complementary elements to disentangle the complex nutrition of a major honey bee parasite. The consumption of hemolymph seems crucial throughout the parasite lifecycle and could provide *V*. *destructor* with essential nutrients. This confirms the results obtained in previous studies [16,17]. Several honey bee proteins were always present in the mite guts and could be vital nutrients that have to be ingested during the parasite feeding. Variations in the quantities of these proteins according to honey bee developmental stages could represent key features in the parasite life cycle used either actively as signals to trigger reproduction or passively as required nutrients for egg production and maturation at a specific stage. Further analyses of the nutrients consumed and on their path inside the mite’s body are crucial and could shed light on key physiological features of *V*. *destructor*, for instance on its ability to uptake undigested specific honey bee protein for its own development.

## Material and methods

### Biological material

Three infested honey bee colonies from Buckfast-Carnica origins were maintained on the University campus (Albi France) and used as *V*. *destructor* providers from March to September 2021. As males do not infest adult honey bees, only mature female mites (also referred to as foundresses) were used in our experiment. They were collected in brood cells from lightly pigmented honey bee pupae (around Day 10 post capping) one hour before the start of the experiment. Six different colonies with low mite infestation levels were used to provide the honey bee stages used in this study from March 2021 to July 2022. Larvae, pupae and adults were collected from brood frames and kept in a Petri dish in an incubator until further use. All of our studies were conducted according to the European ethics laws for scientific research currently in force (Directive 2010/63/EU of the European Parliament and the Council of 22 September 2010 on the protection of animals used for scientific purposes)

### Protocol to maintain starved mite alive for 24 h through artificial PBS feeding

The water source used for the 24 h starvation period consisted of a phosphate buffered saline solution (PBS 1X, Thermo Fisher Scientific, Waltham, MA) coloured with 0.5% of Blue FCF dye solution (Vahiné, Labastidette, France).

To create artificial feeding chambers, the methodology of Posada et al. (2020) [73] was followed, only gelatine capsules were preferred to plastic microcentrifuge tubes. Plastic can indeed be detrimental to the mite [74]. Briefly, a 6 mm large hole was pierced in top halves of 1.37 mL gelatin capsules (LGA, La Seyne sur mer, France). Two breathing holes were pierced with a needle in the bottom half of the capsules before sterilisation for 20 minutes under a UV light. The next steps were conducted under a filtering fume hood in sterile conditions. A piece of Parafilm (4x4cm) was fully stretched to a thickness of 16 μm as in Posada et al. (2020) [73]. A small Parafilm well was then created in the 6 mm hole and 20 μL of feeding solution were deposited inside [44]. A second layer of Parafilm was then used to seal the well and create a bubble containing the feeding solution. Two mites were introduced in the gelatine capsule before being sealed and then transferred in an incubator under standard relative hygrometry (RH) and temperature conditions for *V*. *destructor* maintenance (34°C, 70% RH) [75]. The feeding status of the mites (*i*.*e*. the presence of Blue FCF dye in the gut) and the survival were checked after 24h.

### Natural feeding and sampling protocol for proteomic analyses of honey bee-fed *V*. *destructor*

**Fig 6:**
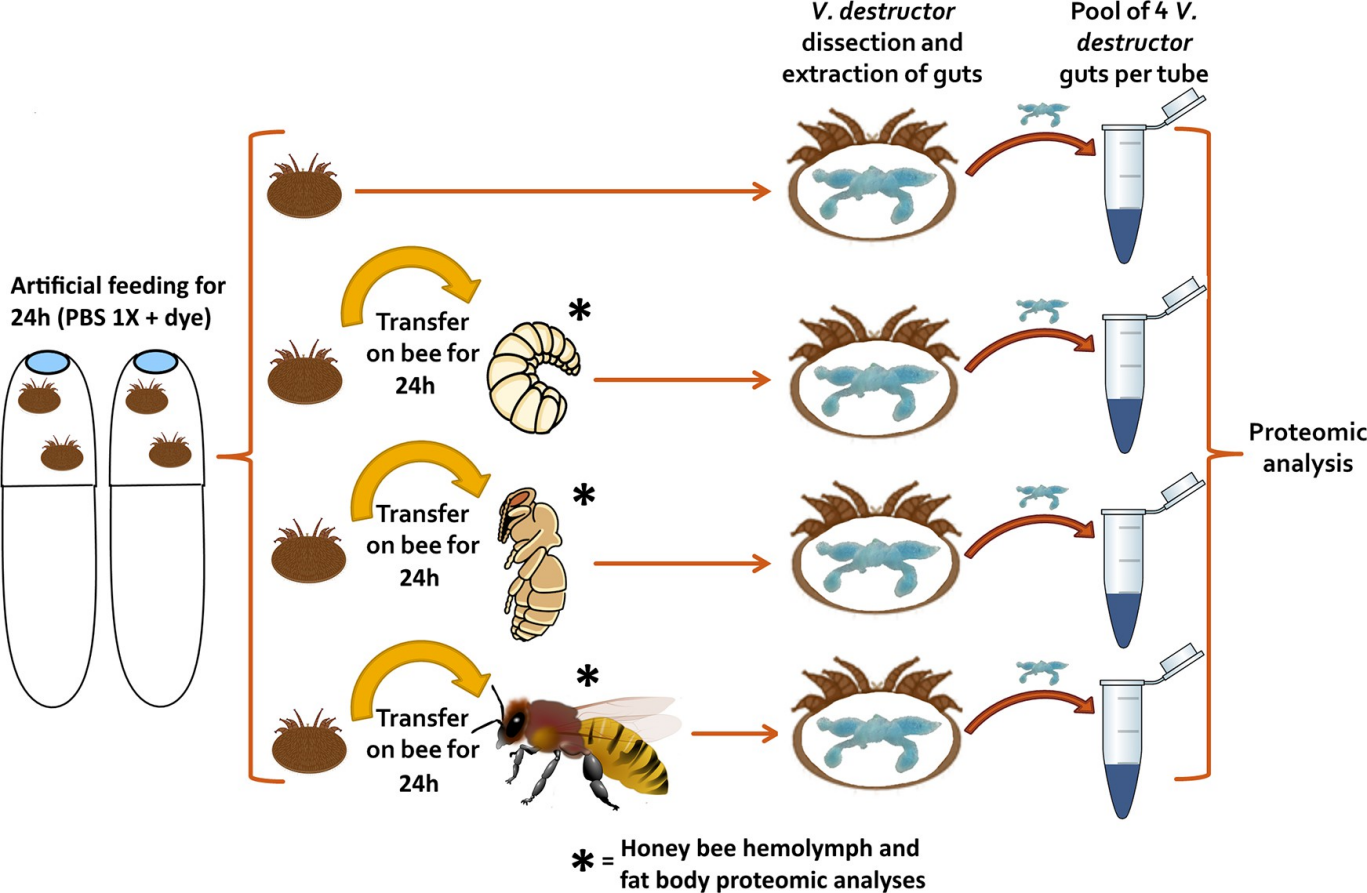
Workflow for the proteomic analyses data collection of mite gut contents. Foundresses *Varroa destructor* were collected from brood frames and transferred in gelatine capsules and starved for 24h on a PBS solution. Four mites from the control conditions were then dissected and their gut was isolated in microcentrifuge tubes. The rest of the mites were transferred onto spinning larvae, pink-eyed pupae or one day old adult bees for 24 additional hours. Four mites per condition (fed on larvae, pupae or adults) were then dissected and their guts pooled in a loBind microcentrifuge tube. Fat body and hemolymph of honey bees of the same developmental stages were also collected and pooled by three (3) in similar tubes. The procedure was repeated three times and all tubes were stored at -40°C until further proteomic analyses. Larva (https://doi.org/10.7875/togopic.2022.304) and pupa pictures (https://doi.org/10.7875/togopic.2022.305) were retrieved from the DataBase Center for Life Science and were conceived by Haru Sakai and Hiromasa Ono.

First, to standardise *V*. *destructor*, the control artificial starvation protocol was followed and the mites left for 24h in an incubator at 34°C and 70% RH (Fig 6). On the next day, the nutritional status and survival of mites were checked, 98% of mites had ingested the coloured solution and 70% were alive. All living mites used in the experiment were thus in the same feeding condition at 24h and can be considered as starved mites. Four of these control starved mites that successfully ingested PBS were then dissected following Piou et al. (2021) [76] and their guts were extracted and pooled in a 0.8 mL loBind microcentrifuge tubes (Eppendorf) with 30 μL of sterile PBS (1X). The rest of the living mites were divided into three test groups that were transferred in 0.68 mL gelatine capsules containing either two spinning larvae, two pink-eyed pupae or two one day old adults (two mites per capsule) [77]. Older living worker honey bees could not be used in our capsule experiments as their high activity results in an increased mortality of mites in this confined environment. The capsules were transferred in an incubator (34.5°C, 70% RH) and further kept for 24h. The female parasites were collected the next day and four mites per condition were dissected. As in the control condition, their guts were extracted and pooled by four in loBind microcentrifuge tubes containing 30 μL of PBS (Fig 6). The tubes were immediately stored at -40°C until proteomic analyses were performed. This procedure was repeated three times on biological material from different colonies to obtain three biological replicates.

To complete the analyses, we compared the gut contents of mites to the honey bee tissues they supposedly consumed. Honey bee hemolymph and fat body were collected on adults, larvae and pupae at the same precise stages as those used in the mite feeding experiment (i.e. spinning larvae, pink-eyed pupae or one day old adults Fig 6). As nurses are more frequently targeted by *V*. *destructor* than emerging bees or foragers [50], the tissues of older adults collected on brood frames were also dissected and analysed to compare their proteomic profiles to the ones of one day old adults used in our study (S4 Table). Briefly, pupae and larvae were disposed in Petri dishes kept on ice and punctured to collect a drop of hemolymph using a micropipette. The hemolymph of adults was sampled by removing the antennae as in Borsuk et al. (2017) [78]. To collect the fat body, the honey bee pupae, larvae and adults were dissected in a PBS buffer using forceps and the fat body tissues were rinsed in PBS before the transfer into the collection tube [79]. Three individuals were pooled in each sample tube and the procedure was repeated three times on each of the three different honey bee colonies. The hemolymph and the fat body samples from the different honey bee stages were stored at -40°C until further proteomic analyses.

### Proteomic analyses

#### Off-gel digestion and nano-LC-ESI-MS/MS, data searching and label free quantification (LFQ)

Three biological replicates from mites (guts pooled from four individuals) or three replicates from each honey bee tissue (hemolymph or fat body pooled from three individuals) were suspended in PBS. The following steps were performed according to Houdelet et al. (2021) [80]. Briefly, one hundred μL of pure HFIP (Sigma-Aldrich, St. Louis, MO) was added to each pool, samples were incubated at 4°C for 4 h prior to an overnight incubation with 0.1% RapiGest surfactant (Waters, Milford, CT). Proteins were reduced with DTT at 56°C in the dark and subsequently alkylated with 4-VP (Sigma-Aldrich, St. Louis, MO) in the dark at RT before being digested with Trypsin (Promega, Madison, WI) at 0.2μg/μL [80] and incubated at 4°C for 4 h under gentle shaking. Digested samples were acidified with a solution of TFA (Carlo-Erba Reagents, Val de Reuil, France) to reach a 0.5% final concentration, to neutralize the buffer and stop the reaction before being incubated for 30 min at 37°C. Finally, samples were dried out under vacuum and resuspended in 2% ACN/0.1% TFA prior to nanoLC-ESI-MS/MS analysis.

#### NanoLC-MS/MS analysis and data acquisition

The tryptic-digested peptides were separated by reversed-phase chromatography using an Ultimate U3000 nano-HPLC equipped with an Acclaim C18 PepMap100 column (3 μm, 75 μm x 250 mm) under conditions reported in Houdelet et al. (2021) [21]. Briefly, separation was performed at a flow rate of 300µL/min using a biphasic linear gradient (water/acetonitrile, each with 0,1% formic acid (Carlo-Erba Reagents, Val de Reuil, France) final concentration) of 2–35% and 35–70% in 120 min and 5 min, respectively. Eluted tryptic peptides were analysed on line by a Q-Exactive Orbitrap mass spectrometer (Thermo Fisher Scientific, MA) in a positive and data-dependant acquisition mode using a scan range 380 to 2000 m/z, as detailed in Houdelet et al. (2021, 2022)[80,81].

#### Database Searching, Annotation, and Label-Free Quantification (LFQ)

Proteome Discoverer version 2.5 (Thermo Fisher Scientific, MA) was used for sequencing and to quantify the proteins’ relative abundance using label-free. A Fasta file protein database containing entries downloaded from NCBI on August 2023 gathered 1,532,988 sequences of hymenoptera, 20,214 protein sequences from *Aethina tumida*, 14,703 sequences from *Tropilaelaps* species, 115,707 entries from mites and 59,574 sequences from *Varroa* species, 7,578 entries from bee viruses, 32,221 entries from *Crithidia* and *Lotmaria* species.

The following parameters were used: trypsin digest with two maximum missed cleavages; six and 150 amino acids as minimum and maximum peptide length, respectively; and a tolerance of 10 ppm/0.02 Da for precursors and fragment ions, respectively. Ethyl-pyridine modification of cysteine was set as a fixed modification; C-terminal protein amidation, methionine and tryptophan oxidation were set as variable modifications. A maximum RT shift of 10 min., a mass tolerance of 10 ppm and a coarse parameter tuning were selected. Protein annotations were successfully validated when the q-value scores were calculated below the false discovery rate (0.05). The protein abundance was determined based on the intensity of the unique and razor peptide precursor ions. The normalisation and the protein roll-up calculation were performed using the total peptide amount (summed abundances) and based on pairwise ratios with a maximum fold change of 100 and a t-test (background based) hypothesis test, without scaling. The modified peptides were not considered for the calculation of the pairwise ratios. The variability of the pairwise ratios was also calculated from all pairwise ratios of all peptides of a protein, as follows: 100 × 1.483 MAD(ratio_1_, …, ratio_m_)/(median_fold_change) where “MAD” is the median absolute deviation, “median_fold_change” is the median_ratio when this is larger or equal to one and 1/median_ratio otherwise. An adjusted p-value was calculated using the Benjamini−Hochberg method and proteins with a p-value less than 0.05 and a fold change ratio of at least 2 were considered as significantly modulated. Based on the protein annotations, the molecular and cellular processes as well as the involved pathways of the quantified proteins were processed using the OmicsBox bioinformatic tool (BioBam Bioinformatics, Spain).

### Statistical analyses

First, a sorting of the initial dataset was performed, for instance to retain only *Apis* spp. proteins or Acari proteins, or to isolate the data from a specific honey bee stage. Graphs and statistical tests were then computed using R (R core team) and the ggplot2 package [82,83]. The survival data over 48h were analysed using a Binomial GLM. Using the dynamic table function in Excel, the *Apis* spp. protein occurrence inside *V*. *destructor* guts was computed as the number of times a particular accession number was detected in our samples. The protein occurrence was analysed using generalised linear models (GLMs) with Poisson distributions (or quasiPoisson in case overdispersion was detected by comparing our data to an artificial Poisson distribution with the same statistical parameters). The fat body and hemolymph protein composition of honey bee larvae, pupae and adults were used as explanatory variables. To check for multicollinearity, the variation inflation factor was calculated and compared to a threshold (commonly fixed at 5). In addition, Kendal correlation analyses between the counts of bee proteins in mite and bee tissues were computed.

Kendal correlation analyses were also run on the quantitative proteomic data. More precisely, significant abundance ratios of *Apis* spp. proteins were analysed in both honey bees and mites to investigate the relation between the variations of protein quantities in honey bee tissues and inside the parasite’s gut throughout the host development. The log transformed abundance ratios and ratio errors of mite’s gut proteins were also calculated to plot graph 3B.

## Supporting information

S1 Fig Number of proteins detected in *V*. *destructor* gut extracts, classified according to their taxonomic origin. The taxonomic origin was divided into Acari, *Apis*, non Apidae hymenoptera, non-hymenopteran insects and bacteria or pathogens.
(PDF)

S2 Fig Survival rate of the mites after 24h and 48h fed on different nutritional solutions. (PDF)

S3 Fig Correspondence Analysis (CA) from the frequency dataset of *Apis* spp. proteins found in the mite gut in relation to its feeding condition during the past 24h. (PDF)

S4 Fig Kendall correlation plots of *Apis* spp. protein counts in *Varroa destructor* gut and honey bee tissues. (PDF)

S5 Fig Comparison of the Log-ratios of protein abundances between *V*. *destructor* guts, honey bee hemolymph or honey bee fat body. (PDF)

S1 Table Most common differentially regulated Acari proteins from fed mite guts compared to starving *V*. *destructor* guts [26–43,85–90]. (XLSX)

S2 Table Proteins of the honey bee are always found in our *V*. *destructor* gut samples and over-represented when mites feed on honey bees compared to starved mites. Proteins highlighted in blue are the proteins that were identified in larva, pupa and adult fed mites.
(XLSX)

S3 Table Identity of the proteins that vary significantly between honey bee stages both in honey bee tissues and in guts of *V*. *destructor* females fed on bees. (XLSX)

S4 Table Variability of *Apis mellifera* proteins between emerging honey bees (one day old) and older adult workers. (XLSX)

S1 Data Initial data set from the qualitative proteomic analysis of mite guts and honey bee tissues. (XLSX)

S2 Data Initial data set from the proteomic LFQ analysis of mite guts and honey bee tissues. (XLSX)
