# Supplementary material for: The proteomic content of Varroa destructor gut varies according to the developmental stage of its host
Source: PLoS Pathog. 2024 Dec 30;20(12):e1012802. doi: 10.1371/journal.ppat.1012802 (PMC11723617; doi:10.1371/journal.ppat.1012802)
Supplement: S1 Fig — The taxonomic origin was divided into Acari, Apis, non Apidae hymenoptera, non-hymenopteran insects and bacteria or pathogens. (PDF) [file ppat.1012802.s001.pdf]

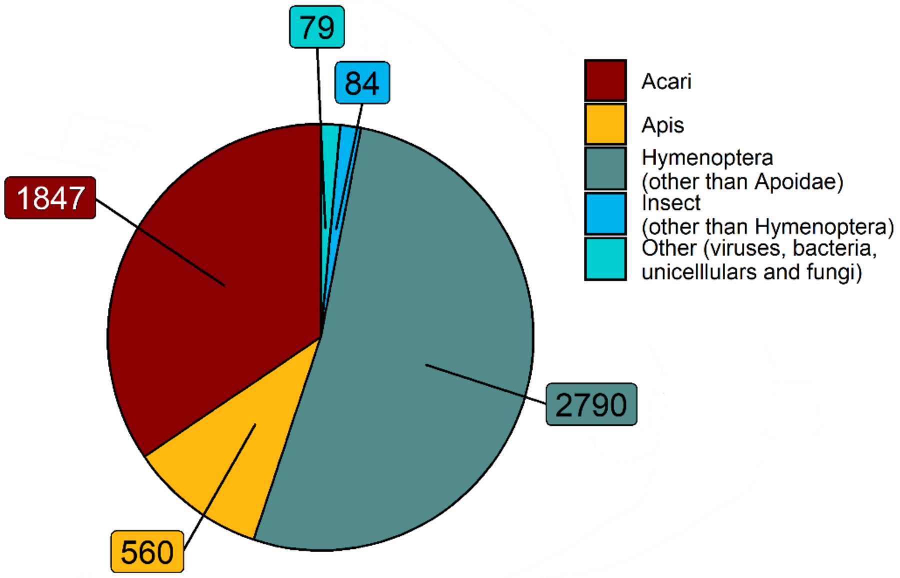

S1 Fig. Number of proteins detected in *V. destructor* gut extracts, classified according to their taxonomic origin. The taxonomic origin was divided into Acari, *Apis*, non Apidae hymenoptera, non-hymenopteran insects and bacteria or pathogens. Of the total of 5,360 proteins identified inside *V. destructor* guts, 34% (1,847) were identified as proteins from Acari, and 64% (3,434) were identified as insect proteins. A large proportion of these 3,434 insect proteins matched to sequences from species of the *Apis* genus (560 or 16.3% of the total insect proteins) or from non *Apis* hymenopteran species (2,790 proteins or 81.2% of total insect proteins). Only 84 proteins (2.5%) matched to sequences from non-hymenopteran insects. In our experimental conditions, we also identified the presence of 79 proteins recognized as originated from different honey bee pathogens or from *V. destructor* microbiome.
