# Supplementary material for: The proteomic content of Varroa destructor gut varies according to the developmental stage of its host
Source: PLoS Pathog. 2024 Dec 30;20(12):e1012802. doi: 10.1371/journal.ppat.1012802 (PMC11723617; doi:10.1371/journal.ppat.1012802)
Supplement: S2 Fig — (PDF) [file ppat.1012802.s002.pdf]

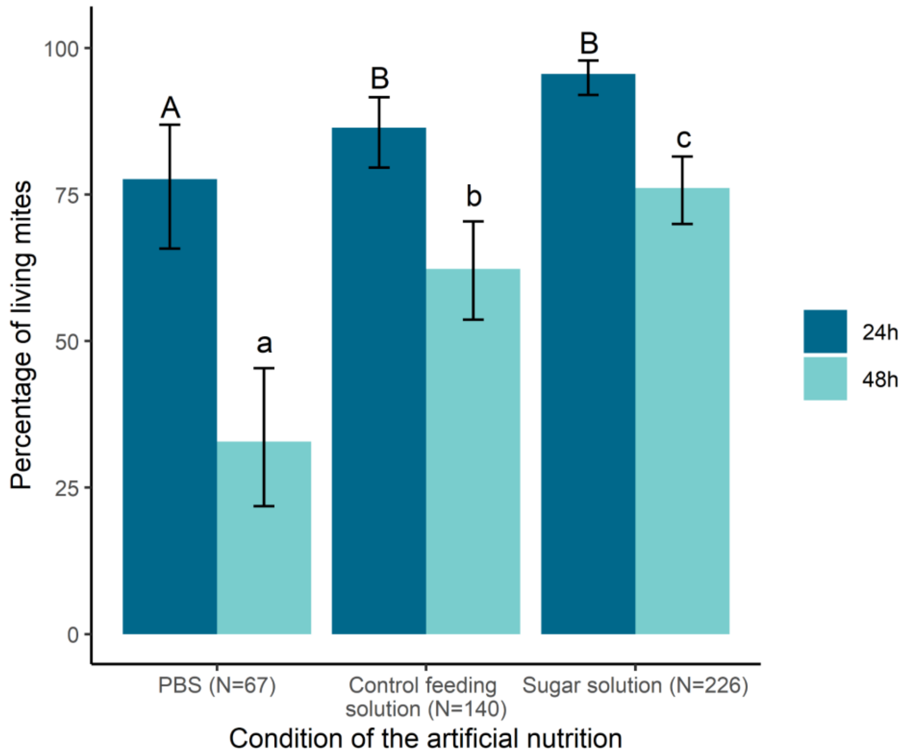

S2 Fig. Survival rate of the mites after 24h and 48h fed on different nutritional solutions, namely PBS, the standard control feeding solutions used in previous studies [1-3] or on a sugar solution composed of 50 mg/ml fructose and 50 mg/ml glucose. The differences observed were significant for both times (24h: χ^2=20.23, df=2, p<0.001; 48h: χ^2=42.08, df=2, p<0.001). After 24h, the mites survived more often when fed on a sugar or standard solution (p=0.25) than on PBS (adjusted p<0.01 and 0.001, respectively). At 48h, all conditions were significantly different and the survival was slightly higher for a sugar solution compared to a standard solution (adjusted p<0.05) or much higher compared to PBS (adjusted p<0.001). N represents the numbers of individuals tested.

References

[1] Posada-Florez F, Ryabov E, Heerman MC, Chen Y, Evans JD, Sonenshine DE, et al. *Varroa destructor* mites vector and transmit pathogenic honey bee viruses acquired from an artificial diet. Plos One. 2020;15: 1–13. doi:10.1371/journal.pone.0242688

[2] Bruce WA, Chiesa F, Marchetti S, Griffiths DA. Laboratory feeding of *Varroa Jacobsoni* Oudemans on natural and artificial diets (Acari: Varroidae). Apidologie. 1988;19: 209–218.

[3] Tabart J, Colin ME, Carayon JL, Tene N, Payre B, Vetillard A. Artificial feeding of *Varroa destructor* through a chitosan membrane: A tool for studying the host-microparasite relationship. Exp Appl Acarol. 2013;61: 107–118. doi:10.1007/s10493-013-9675-9
