# Supplementary material for: The proteomic content of Varroa destructor gut varies according to the developmental stage of its host
Source: PLoS Pathog. 2024 Dec 30;20(12):e1012802. doi: 10.1371/journal.ppat.1012802 (PMC11723617; doi:10.1371/journal.ppat.1012802)
Supplement: S3 Fig — (PDF) [file ppat.1012802.s003.pdf]

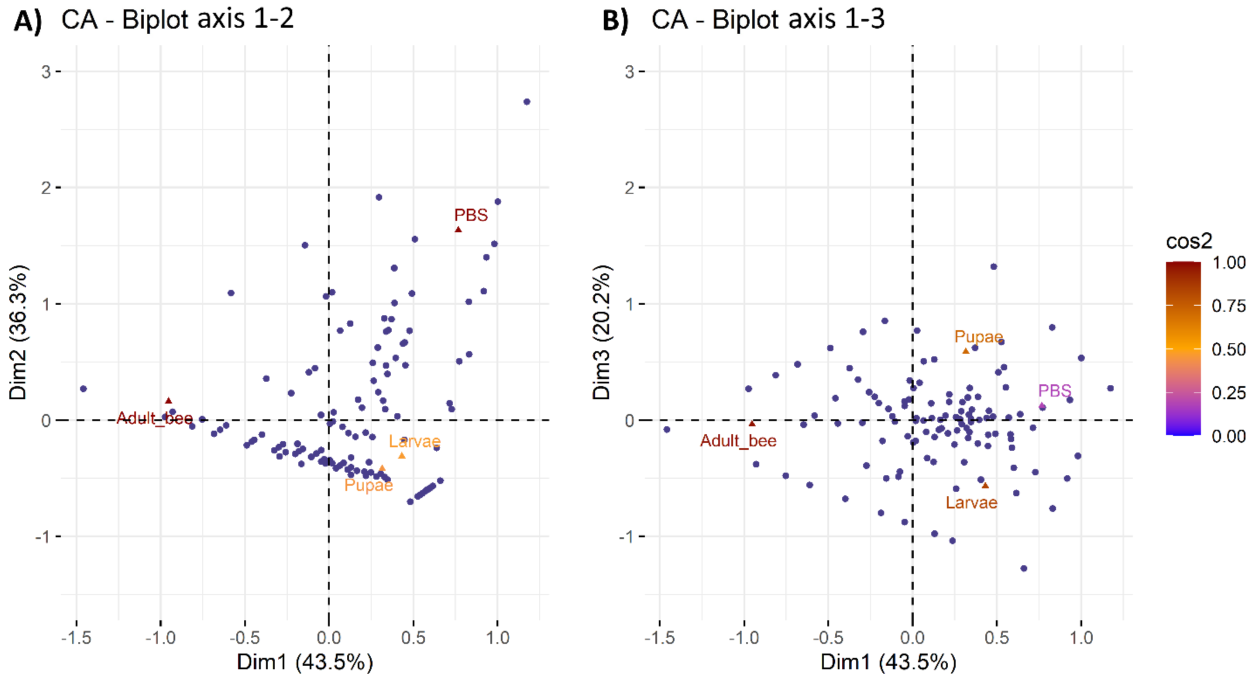


**S3 Fig. Correspondence Analysis (CA) from the frequency dataset of *Apis* spp. proteins found in the mite gut in relation to its feeding condition during the past 24h**. (A) Visualization of axis 1 and 2, (B) Visualization of axis 1 and 3. Mites fed on pupae and larvae shared a more similar bee protein profile between each other than mites fed on adults. The PBS fed group is also different from the three other groups.
