# Supplementary material for: The proteomic content of Varroa destructor gut varies according to the developmental stage of its host
Source: PLoS Pathog. 2024 Dec 30;20(12):e1012802. doi: 10.1371/journal.ppat.1012802 (PMC11723617; doi:10.1371/journal.ppat.1012802)
Supplement: S4 Fig — (PDF) [file ppat.1012802.s004.pdf]

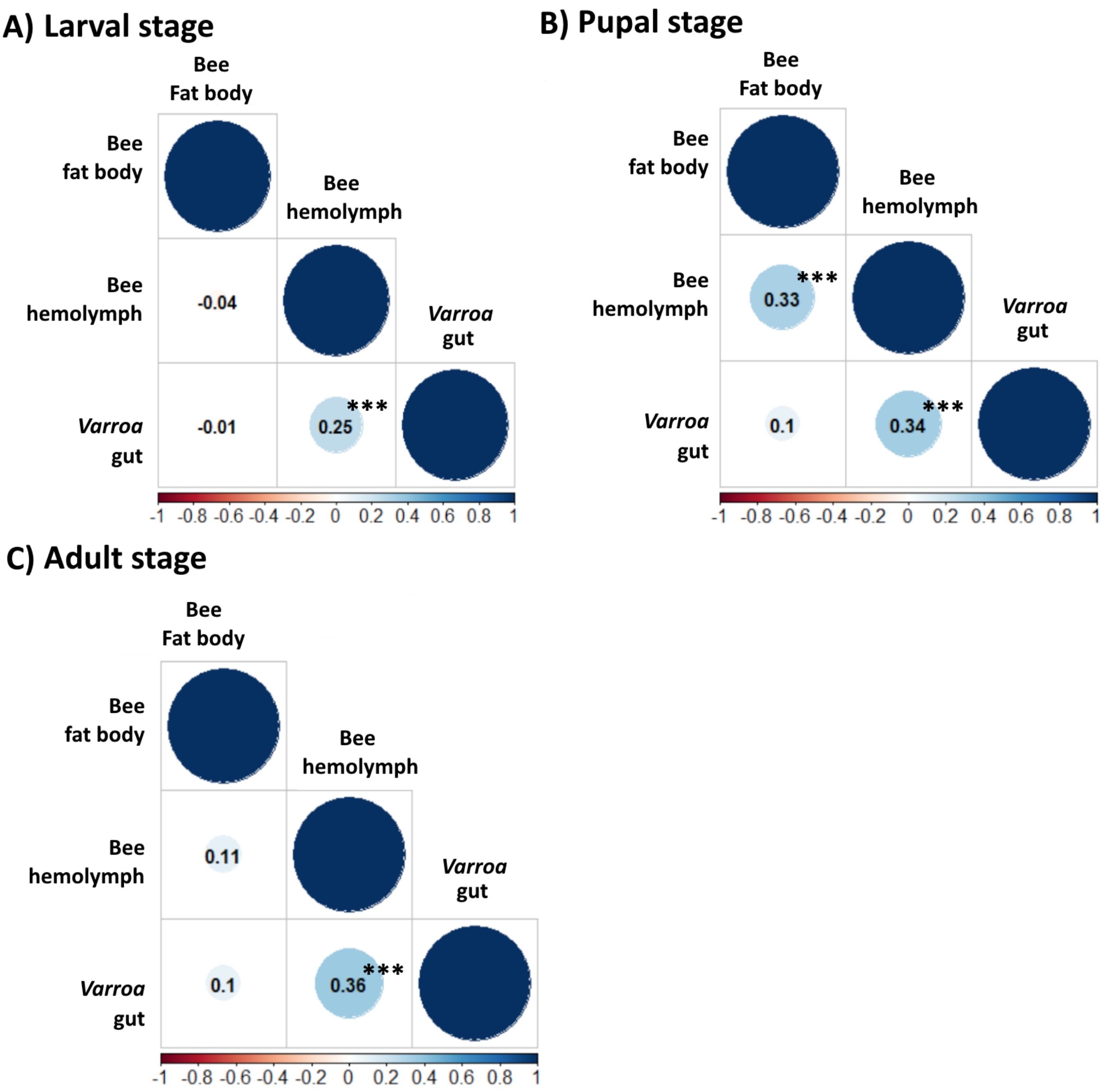

S4 Fig. Kendall correlation plots of *Apis* spp. protein counts in *Varroa destructor* gut and honey bee tissues.

(A) Proteins found in bee larvae and larva-fed groups; (B) Proteins found in bee pupae and pupa-fed mites; (C) Proteins found in adult bees and adult-fed mites. Kendall τ are shown inside circles and asterisks indicate the significance after Bonferroni corrections ($p<0.001$).
