# Supplementary material for: The proteomic content of Varroa destructor gut varies according to the developmental stage of its host
Source: PLoS Pathog. 2024 Dec 30;20(12):e1012802. doi: 10.1371/journal.ppat.1012802 (PMC11723617; doi:10.1371/journal.ppat.1012802)
Supplement: S5 Fig — (PDF) [file ppat.1012802.s005.pdf]

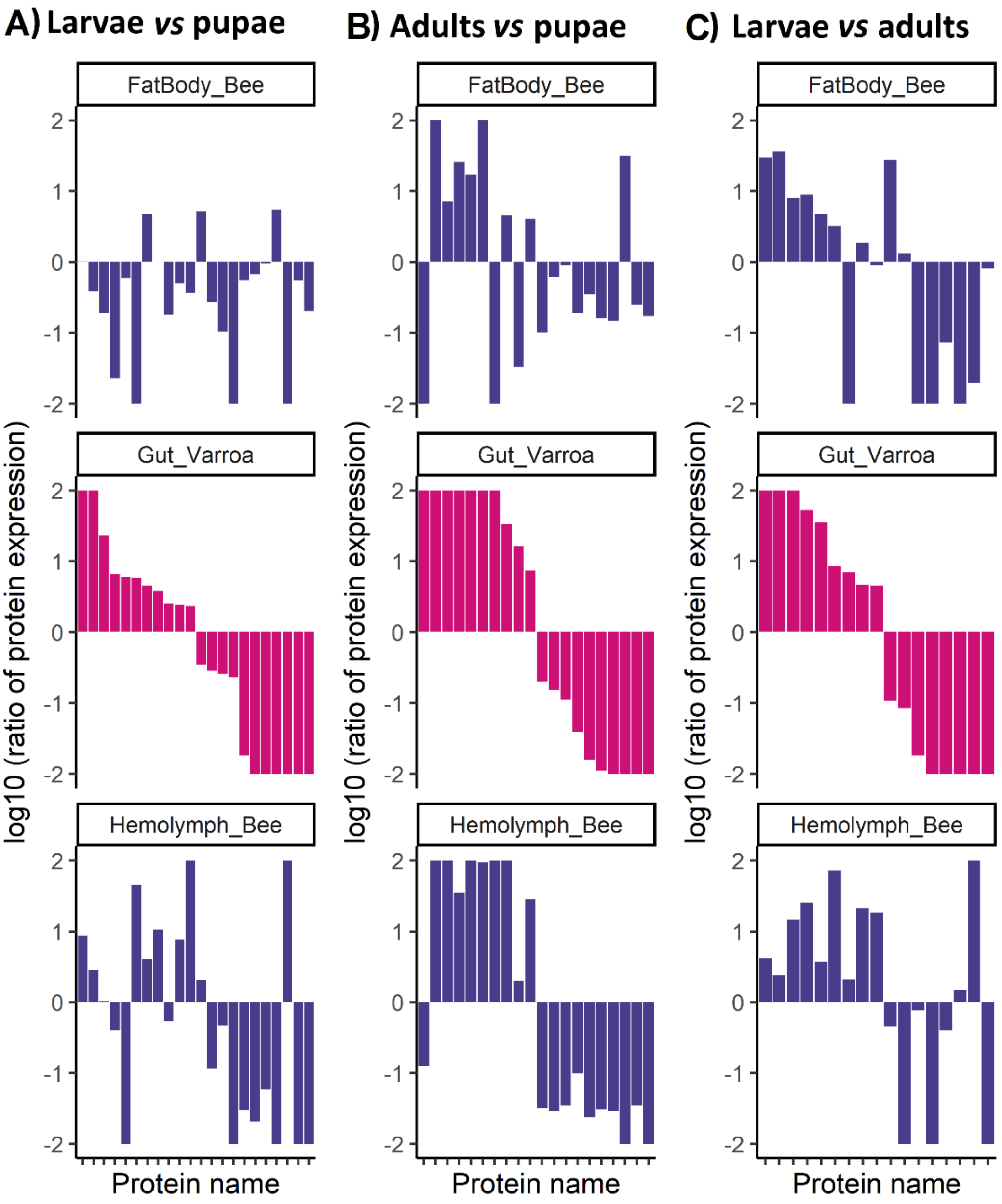

S5 Fig. Comparison of the Log-ratios of protein abundances between *V. destructor* guts, honey bee hemolymph or honey bee fat body.

Three pairwise ratios could be calculated according to the developmental stages compared, namely (A) Ratios between larvae and pupae, (B) Ratios between adults and pupae and (C) Ratios between larvae and adults. Each protein from the mite gut (middle graph) is represented by a bar and aligned vertically with the same protein found in the honey bee fat body (top graph) or hemolymph (bottom graph). The variable proteins highlighted through quantification are detailed in the S3 Table. If the mite feeds on one tissue in particular, the general aspects of the protein variations should be reflected inside the *V. destructor* gut.
